# The Teaching of Operative Surgery

**Published:** 1912-12-21

**Authors:** 


### THE MAKING OF THE MODERN PRACTITIONER.

### The Teaching of Operative Surgery.

At the present time there is in this country, for all practical purposes, one royal road to success as an operator—to success, that is, in its material or commercial aspect. That method is to secure an appointment on the visiting surgical staff of a voluntary hospital, preferably of a general hospital with a medical school attached to it. He who adopts this plan devotes all his energies to the attainment of such a post; as a rule this entails a probationary period of some few years, during which he coaches students for examinations and acts as surgical registrar, demonstrator of anatomy, resident surgical officer, or any other transitional appointment which will serve the double purpose of affording him surgical experience and of bringing him to the favourable notice of his senior colleagues.

### Why the Best Surgeons are not Elected.

It would appear at first sight as if this system, haphazard as it undoubtedly is, still has the merit of securing the election to the staff of our teaching hospitals of the very cream of the surgical talent of the day. But a variety of reasons conspire to impede such a happy consummation. The general level of efficiency is certainly a very high one; but it might be higher than it is, if only the method of selection could be amended in certain particulars.

It may be objected that no adequate proof has been adduced of the thesis just advanced—namely, that the best surgeons do not always get elected to the staff of the teaching hospitals. But no one who is really intimate with the affairs of any medical school would care to dispute it; and if anyone did so, he could be easily confronted with a long list of surgeons, physicians, and obstetricians of the highest eminence who were rejected by their original schools and teachers.

In respect of surgery, there are many difficulties. Practically all the offices which young men, who aspire to surgical distinction, can fill as a preliminary to election as assistant surgeons afford them very little chance of operating. So that a new assistant surgeon is nearly always elected, not because he is known to be a good operator (or likely to become one), but because he has done well as a student, been tactful as a resident, and taught well as a demonstrator of anatomy. It thus happens that a number of men become assistant surgeons, and in due time surgeons, on the staffs of important hospitals, who are in fact less well qualified for their responsibilities than others who have had to go without such opportunities.

It is safe to say that every well-known teaching hospital has one such square peg in a round hole; and lucky is that institution which has one and no more. It would be invidious, possibly libellous, to mention names; and there is no need to do so, since the fact is entirely beyond dispute.

Another factor which tends towards the same result is this. The long probationary period of doing odd jobs, and the often longer round of out-patient work before consulting practice comes, may and often do prevent a man who has no private means from entering the competition. Many a country practitioner has in him the making of a fine surgeon; but has had to stifle ambition through the pressing necessity of setting forth to earn money as soon as he has been qualified, or at least as soon as he has quitted his resident appointments.

Dr. G. E. Herman, the well-known consulting gynæcologist to the London Hospital, offers some suggestions in a recent issue of the *Practitioner*, which may have the effect, if adopted, of improving the state of things to which attention has just been called.

### A Gynæcologist's Suggestion.

He criticises severely the usual classes in operative surgery, stating quite truly that the operations there done on the dead subject are just those which modern antiseptic surgery has rendered quite obsolete, while the hundreds of others which are now of widespread value are still not practised at all in the classes. To remedy this state of things he quotes with approval a plan which has been tried or suggested in America. Each pupil at an operative surgery class is provided with a live pig. On this he performs some operation, say, gastro-enterostomy, which does the animal no harm: the operation is, of course, done under an anæsthetic. Full antiseptic and aseptic precautions have to be taken, otherwise the pig will die; and thus the exact technique of abdominal operating is easily taught. A good surgeon can do twenty or more such operations on a pig at short intervals; whereas a bad one would probably lose five or six or more animals. The pig is the animal chosen because its intestines resemble those of man more nearly than do those of other animals. Dr. Herman argues very ably that this is the proper system of instruction; and it can scarcely be doubted that he is right. Not only so, this system would enable the man with real surgical instincts to come to the front much more rapidly and certainly than at present; and we look forward with great interest to some experiment along these lines in Britain.

In the same article Dr. Herman renews a plea which he has often raised before. He contends that the system by which the assistant surgeons see out-patients and the older men do the operating is wrong. He would have the young surgeons for the in-patients and their seniors for out-patients. Like everything Dr. Herman writes of, this contention is presented with great force. We doubt whether this reform is ever likely to be realised; but, as a matter of fact, the junior surgeons do now get far more opportunities of operating than their predecessors were accustomed to. And this is true not only of the London Hospital, but of many others both in the Metropolis and the provinces; and the tendency is one to be strongly encouraged.

